# The impact of a physician detailing and sampling program for generic atorvastatin: an interrupted time series analysis

**DOI:** 10.1186/s13012-017-0671-z

**Published:** 2017-11-25

**Authors:** Heather C. Worthington, Lucy Cheng, Sumit R. Majumdar, Steven G. Morgan, Colette B. Raymond, Stephen B. Soumerai, Michael R. Law

**Affiliations:** 1 Centre for Health Services and Policy Research, School of Population and Public Health, Faculty of Medicine, The University of British Columbia, Vancouver, British Columbia Canada; 2 Department of Medicine, University of Alberta, Edmonton, Alberta Canada; 3 School of Population and Public Health, Faculty of Medicine, The University of British Columbia, Vancouver, British Columbia Canada; 4 Manitoba Centre for Health Policy, Max Rady College of Medicine, Rady Faculty of Health Sciences, University of Manitoba, Winnipeg, Manitoba Canada; 5 Department of Population Medicine, Harvard Medical School, Boston, MA USA; 6 201-2206 East Mall, Vancouver, BC V6T 1Z3 Canada

**Keywords:** Detailing, Atorvastatin, Sampling, Generic drugs

## Abstract

**Background:**

In 2011, Manitoba implemented a province-wide program of physician detailing and free sampling for generic atorvastatin to increase use of this generic statin. We examined the impact of this unique combined program of detailing and sampling for generic atorvastatin on the use and cost of statin medicines, market share of generic atorvastatin, the choice of starting statin for new users, and switching from a branded statin to generic atorvastatin.

**Methods:**

We conducted a retrospective study of Manitoba insurance claims data for all continuously enrolled patients who filled one or more prescriptions for a statin between 2008 and 2013. Data were linked to physician-level data on the number of detailing visits and sample provision. We used interrupted time series analyses to assess policy-related changes in the use and cost of statin medicines, market share of generic atorvastatin, the choice of starting statin for new users, and switching from a branded statin to generic atorvastatin.

**Results:**

The detailing program reached 31% (651/2103) of physicians who prescribed a statin during the study period. Collectively, these physicians prescribed 61% of statins dispensed in the province. Free sample cards were provided to 61% (394/651) of the detailed physicians. The program did not change the level or trend in the overall statin use rate and the total cost of statins or increase the number of patients switching from another branded statin to generic atorvastatin. We found the program had a small impact on atorvastatin’s market share of new prescriptions, with a level increase of 2.6%.

**Conclusions:**

Though physician detailers were skilled at targeting high-prescribing physicians, a combined program of detailing visits and sample provision for generic atorvastatin did not lower overall statin costs or lead to switching from branded statins to the generic. The preceding introduction of generic atorvastatin appeared sufficient to modify prescribing patterns and decrease costs.

## Background

Controlling prescription drug expenditures remains a top priority for both public and private drug programs. One important cost-saving opportunity is increasing the use of cheaper (but therapeutically interchangeable) generic alternatives [1]. In Canada, each province has its own public drug plan and policies regarding generic drugs. However, the use of generics is encouraged by all Canadian provincial drug plans and some private drug plans through some form of mandatory generic substitution rules and interchangeability provisions that allow generics to be dispensed instead of more expensive brand-name alternatives [2]. As a result, generic drugs represented 71.5% of all the prescriptions dispensed in Canada in 2013 [3]. It is in the interest of insurers worldwide to encourage switching to generic alternatives given the major potential for savings, especially as the availability of generic drugs continues to rise [4].

One commonly used and effective method to change prescribing is “physician detailing”—the use of face-to-face visits by sales representatives to promote the prescribing of particular medicines [5, 6]. Most frequently, methods are employed to increase the use of medicines remaining under patent protection. However, many drug programs have attempted to steer physicians toward prescribing the most cost-effective agent using similar tactics of face-to-face visits in the practice of academic detailing, in order to improve both quality and cost effectiveness of care [7].

While the impact of academic detailing in isolation has been well studied [8], there have been few rigorous studies on the impact of providing samples on physician-prescribing behavior. The small number of published studies suggests that the availability of branded samples increases prescribing of more expensive branded medications over more cost-effective and/or preferred medications [9–12]. Two observational studies suggest that generic sampling programs on their own have a small or limited impact on overall generic dispensing rates [13, 14]. To our knowledge, there has only been one study pairing generic sampling with physician detailing that demonstrated a 1.77% increase in the overall generic dispensing rate over and above the impact of physician detailing alone [15]. We are not aware of any studies that compare a combined program of physician detailing and sampling to the status quo (no intervention).

In 2011, Manitoba Health implemented an innovative program aimed at increasing the use of generic atorvastatin, a cholesterol-lowering statin medicine. At the time, atorvastatin was the top-selling drug in the world [16]. Many other drugs are also available within the statin class (such as rosuvastatin, simvastatin, and fluvastatin) and are therapeutically equivalent [17]. This program used detailers who visited high-prescribing physicians to promote the use of generic atorvastatin, with the intent of encouraging new patients to start on generic atorvastatin and established patients to switch to generic atorvastatin from rosuvastatin, which at the time remained available only as a more expensive branded drug. What differentiated this program from conventional “academic detailing” is that it included the distribution of cards that provided free samples of generic atorvastatin to physicians. As there are no rigorous data on the efficacy of a combined program of detailing visits and sample provision on generic drug use, we studied the impact of the program.

## Methods

### Study context

In 2011, Manitoba was the fifth-most populous province in Canada, with a population of 1.2 million [18]. Manitoba has a provincial Pharmacare program that covers all Manitobans; however, drugs are only reimbursed after a patient pays an annual deductible based on family income [19]. In this system, prescription drugs are paid for by a mix of public and private coverage, as well as out-of-pocket by the patient.

The province of Manitoba granted an exclusive listing on the public formulary to the generic manufacturer Ranbaxy for atorvastatin in June 2010 [20]. Reimbursement of the Ranbaxy version of atorvastatin (Ran-Atorvastatin®) by the public plan began on October 14, 2010. In exchange for this exclusive listing, Ranbaxy initiated a program of physician detailing and free sampling, which started in June 2011. Ranbaxy used a private company specializing in the promotion of medicines and recruited two staff members to detail physicians. These representatives promoted the use of Ran-Atorvastatin® to physicians that were high prescribers of statins in the province and provided electronic cards that patients could exchange at the pharmacy for a free 30-day supply of Ran-Atorvastatin®. The program aimed to increase Ran-Atorvastatin® prescribing while decreasing prescribing of more expensive but therapeutically equivalent branded atorvastatin and branded rosuvastatin. As a private firm conducted these visits, we assumed that the detailing methods employed were similar to those used for detailing visits of other medicines. The intent was that these representatives should detail physicians every 45 to 60 days, with a target to see each physician six to nine times per year.

### Data sources and study population

We conducted a retrospective study of Manitoba insurance claims data for all continuously enrolled patients who filled one or more prescriptions for a statin between June 2008 and March 2013. This includes data from 2 years prior to generic atorvastatin being available on the Manitoba Pharmacare formulary until 21 months following the implementation of the generic detailing and sampling program. Based on prescriber information for each statin prescription, we linked this insurance claims data to physician-level information on the date of each detailing visit and the number of sample cards provided during each encounter.

This study used administrative data that includes individual-level data on virtually all Manitobans contained in the Population Health Research Data Repository, which is housed at the Manitoba Centre for Health Policy [21]. Four population-based, administrative data sources were used in this analysis and linked using anonymous identifiers: (1) prescription dispensation records from outpatient dispensaries through Manitoba Health’s Drug Programs Information Network, (2) Manitoba Health Population Registry, (3) Manitoba Provider Registry (prescribers), and (4) records on the physician detailing visits made to each physician.

### Study cohorts

To study the impact of the detailing and sampling program on particular groups, we constructed two sub-cohorts of patients according to the following definitions:

1. *Switching*: “Switchers” received a prescription for a statin after the receipt of two or more prescriptions for a different statin (either a different brand or a brand vs. generic) over the past year.
2. *Starting*: “Starters” received a prescription for a statin after receiving no statin prescriptions in the previous year [22].

### Hypotheses

We hypothesized that the program would increase the use of generic atorvastatin, decrease the use of other statins, and decrease total costs; that the policy would have a greater impact on new starters than those switching from a branded statin to generic atorvastatin; and that the policy would promote more switching from branded to generic atorvastatin than from branded rosuvastatin to generic atorvastatin.

### Statistical analysis

We used interrupted time series analysis to study longitudinal changes in drug utilization and costs, the statins which patients start on, and rates of switching [23]. This method has the distinct advantages of being methodologically rigorous and easily interpretable by non-technical audiences while also controlling for pre-existing secular trends in the outcome. It has been successfully used by many previous pharmaceutical policy evaluations in Canada [24–27]. Using interrupted time series analysis, we were able to estimate the change in the level and the trend of each outcome following the start of the detailing and sampling policy. As the monthly observations were correlated over time, we controlled for autocorrelation using appropriate adjustments in a generalized least squares model [23].

Our analyses used the following measures to determine the impact of the policy:

#### Prescription drug utilization

Our analysis of drug utilization focused on the number of prescriptions. In this analysis, we examined whether the detailing and sampling program increased the overall level of statin prescribing in the province or led to changes between different types of statins. For this analysis, we classified drugs into six groups: (1) branded atorvastatin, (2) generic atorvastatin, (3) branded rosuvastatin, (4) generic rosuvastatin, (5) other branded statins, and (6) other generic statins. Branded drugs, such as Lipitor® in the case of atorvastatin and Crestor® in the case of rosuvastatin, are released by the company that originally developed and marketed the medicine. Generics are drugs of the same molecule that come to market after patent protection expires.

#### Costs

We examined the impact of the detailing program on statin cost by studying the longitudinal change in overall statin costs within the province.

#### Switching rate

Among switchers, we examined longitudinal changes in the proportion of patients that switched from (1) branded atorvastatin to generic atorvastatin and (2) branded rosuvastatin to generic atorvastatin.

#### Starting medication

Amongst starters, we examined longitudinal changes in the proportion of patients initiated on each of the six types of statins.

## Results

### Program reach

The detailing program reached 31% (651/2103) of physicians who prescribed a statin during the study period. Collectively, these detailed physicians prescribed 61% of statins dispensed in the province. Between June 2011 and February 2013, 5154 detailing visits were conducted, with the average physician being visited 7.9 times (approximately once every 3 months). The maximum number of visits to one physician was 30. Free sample cards were provided to 61% (394/651) of the detailed physicians. Of those physicians provided with sample cards, the median number of times sampled (visits during which sample cards were provided) was two and the maximum number of times sampled was 14.

### Study cohort

There were 152,020 statin users in Manitoba with continuous coverage between 2008 and 2013 included in the study. Of these users, 66,091 (43.4%) were new users and 69,876 (46.0%) switched statins over the study period. The age and sex of the study cohort, as well as the new starter and switcher cohorts, are presented in Table 1.

**Table 1:** Age and sex of statin users, and new starter and switcher cohorts of statin users

Age		≤ 44 (%)	45–54 (%)	55–64 (%)	65–74 (%)	75+ (%)	All ages
Users	Female	5.2	15.6	28.8	25.2	25.2	70,535
	Male	8.3	20.5	30.8	16.6	23.8	81,484
Starter	Female	8.7	21.9	32.5	15.5	21.4	30,401
	Male	13.2	26.7	30.8	10.3	19.0	35,690
Switcher	Female	3.5	14.0	29.1	26.5	26.9	31,818
	Male	5.7	18.7	32.2	17.8	25.6	38,058

### Overall statin prescribing

**Fig. 1:**
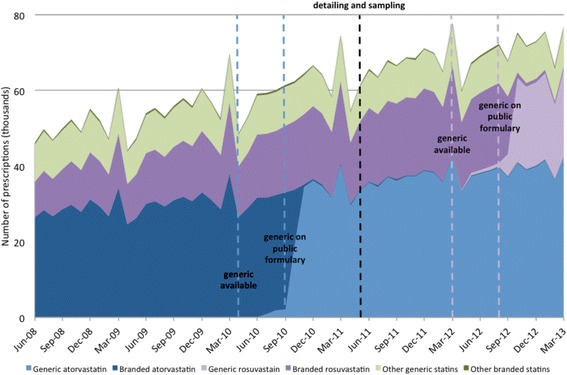
Statin prescribing in Manitoba from 2008 to 2013

We observed that overall statin prescribing increased steadily by 486 prescriptions per month over the study period (95% CI 408 to 565, *p* < 0.01, Fig. 1). When generic atorvastatin became available, there was an almost complete replacement of branded atorvastatin within a few months. We observed a similar pattern for generic rosuvastatin. However, neither the release of generic atorvastatin (level change of 732 prescriptions, 95% CI − 2223 to 3686, *p* = 0.28, trend change of − 40 prescriptions per month, 95% CI − 574 to 494, *p* = 0.74) nor the detailing and sampling program (level change of 785 prescriptions, 95% CI − 1897 to 3467, *p* = 0.20, trend change of − 156 prescriptions per month, 95% CI − 695 to 384, *p* = 0.21) had a significant impact on overall statin prescribing.

### Overall statin costs

**Fig. 2:**
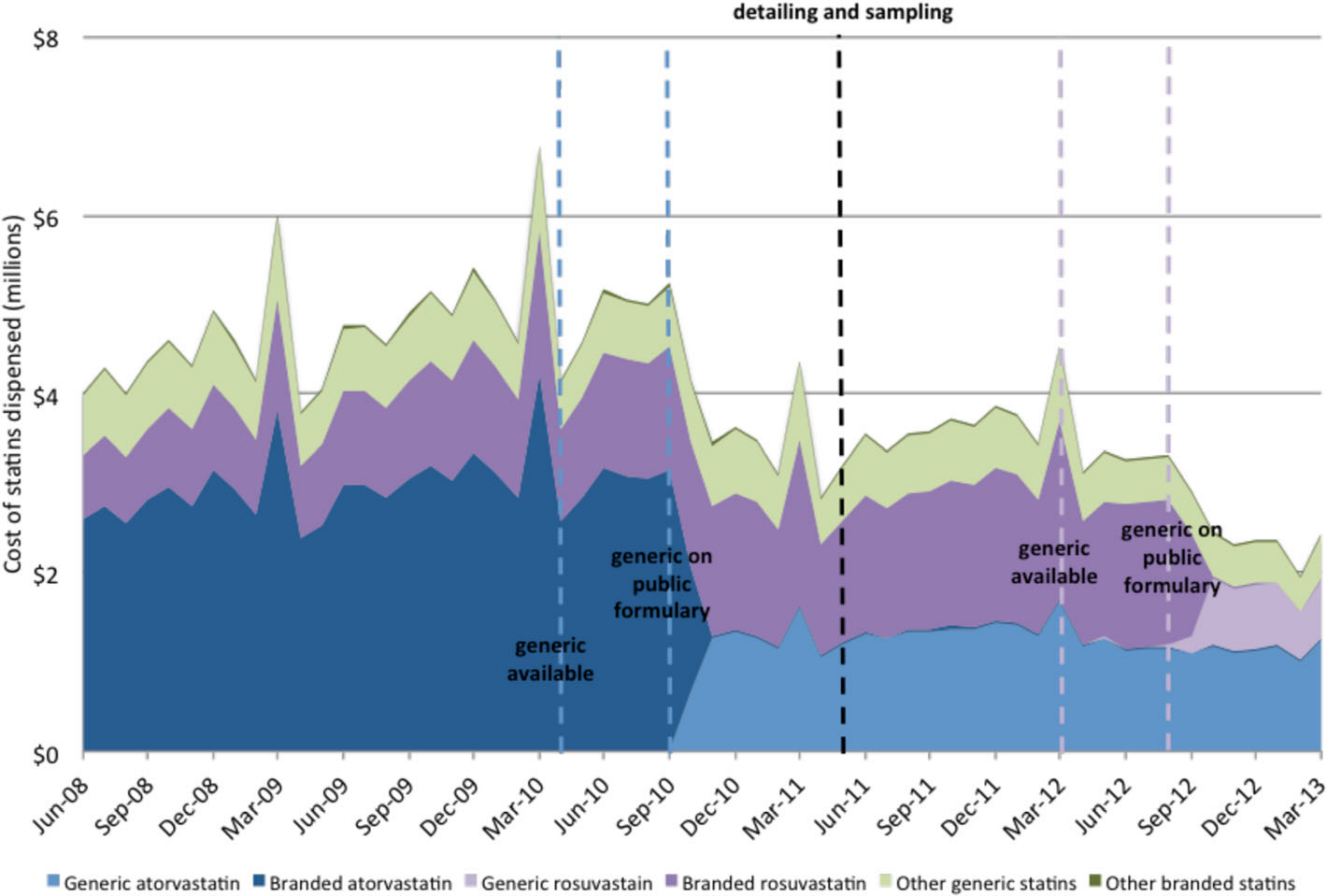
Total cost of statins in Manitoba from 2008 to 2013

Total statin costs were rising at a rate of $34,884 per month (95% CI 18,325 to 51,443, *p* < 0.01) before the introduction of generic atorvastatin (Fig. 2). When generic atorvastatin was put on the Manitoba formulary, there was a significant $1,096,543 drop in cost of statins (95% CI − 1,720,604 to − 472,482, *p* < 0.01), though we found no significant change in trend ($− 81,721 per month, 95% CI − 194,527 to 31,086, *p* = 0.25). We found no further changes in the level ($307,228, 95% CI − 259,149 to 873,604, *p* = 0.32) or trend ($− 38,513 per month, 95% CI − 152,474 to 75,449, *p* = 0.60) of statin costs after the implementation of the detailing and sampling program.

### Switchers

**Fig. 3:**
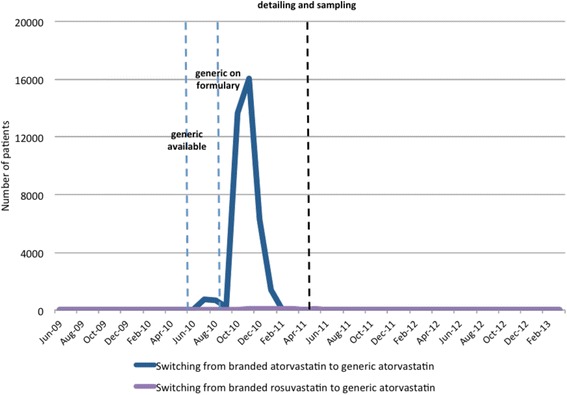
Number of people who switched from a branded statin to generic atorvastatin from 2009 to 2013

When generic atorvastatin was put on the Manitoba formulary, 37,614 patients switched from branded atorvastatin to generic atorvastatin in the 6 months following. In contrast, we found no meaningful change in the number of patients switching from either branded atorvastatin or branded rosuvastatin to generic atorvastatin following the start of the detailing and sampling program. This can be seen in Fig. 3.

### Starters

**Fig. 4:**
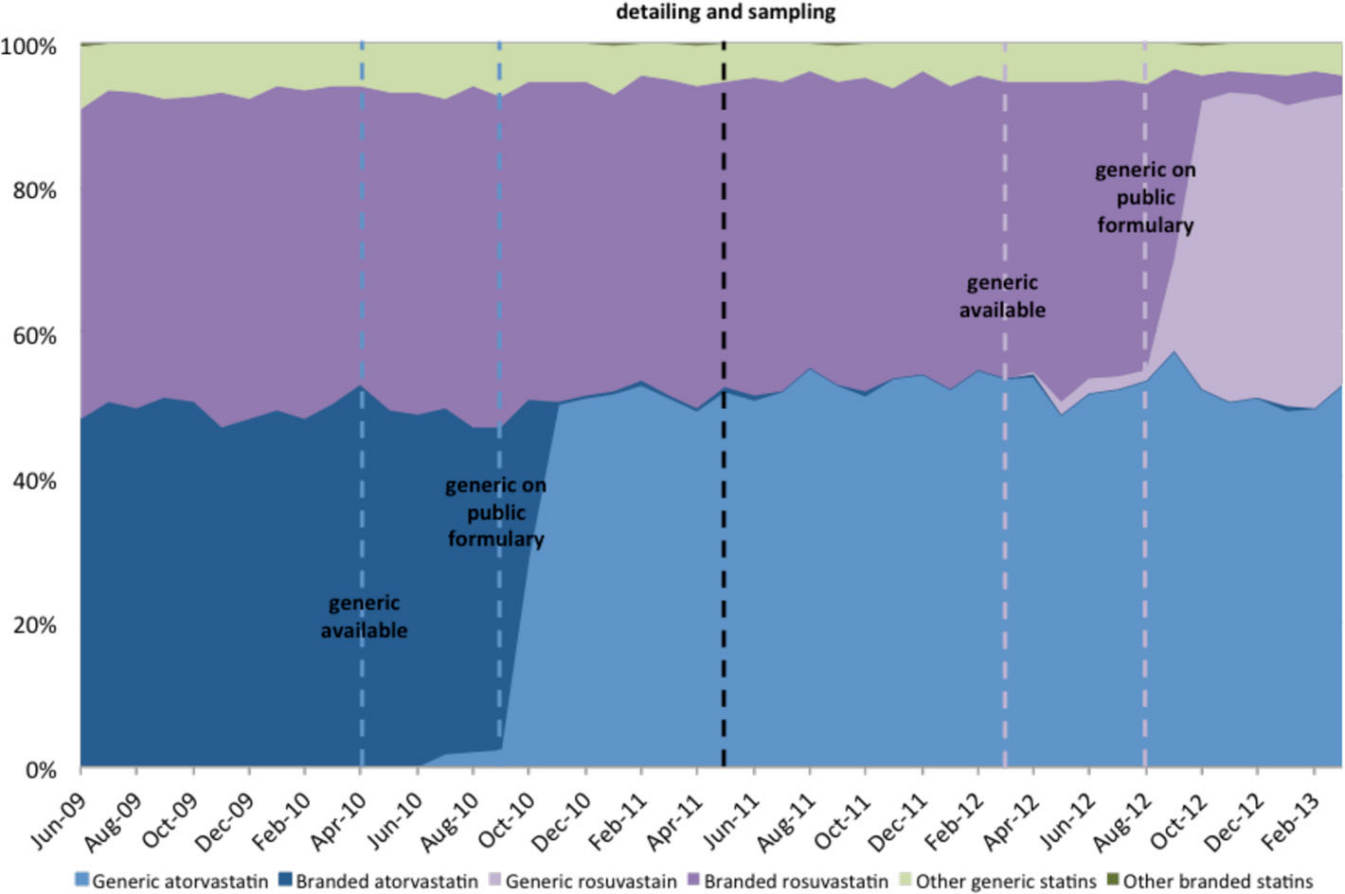
Percent market share of new statin prescriptions in Manitoba from 2009 to 2013

Prior to the introduction of generic atorvastatin, approximately 1560 patients were newly started on statins each month. As shown in Fig. 4, the introduction of generic atorvastatin did not impact the level (1.3%, 95% CI − 1.3 to 3.9%, *p* = 0.11) or trend (0.1% per month, 95% CI − 0.4 to 0.6%, *p* = 0.42) of atorvastatin’s share of new statin prescriptions. We did find, however, that the detailing and sampling program had a small impact on atorvastatin’s market share of new prescriptions, with an increase of 2.5% (95% CI 0.1 to 5.0%, *p* < 0.01). Overall, this change translates to approximately 126 more new atorvastatin users in the year following the program than would have been expected based on existing trends. There was no significant impact on the trend of atorvastatin’s market share of new prescriptions following the detailing and sampling program (− 0.2% per month, 95% CI − 0.7 to 0.3%, *p* = 0.14).

## Discussion

As a growing number of commonly prescribed drugs lose patent protection, it is becoming increasingly important to promote the use of less expensive generic medicines. Despite being run by a commercial detailing firm and having successfully targeted high-prescribing physicians, we found that the use of physician detailing and sample provision in Manitoba did not meaningfully impact either generic statin use or overall statin costs. Importantly, we found the program did not promote switching established users from branded rosuvastatin, despite this being one of the main aims of the program. Finally, while we did find an impact on the choice of starting statin for new users, the size of this effect was very small.

Our results run counter to a significant body of evidence in support of academic detailing [7, 28–33] and mounting evidence in support of sample provision in modifying prescribing behavior [9–11]. We propose two possible explanations for this discrepancy. First, mandatory generic substitution rules and interchangeability provisions existed in Manitoba that required pharmacists to dispense generics in place of equivalent brand-name alternatives [34]. This means that even if a physician prescribes the branded drug, unless the physician has explicitly stated that there are to be no substitutions, the pharmacy will dispense the generic drug. As shown above, when generic atorvastatin became available on the Manitoba formulary, there was an almost complete replacement of branded atorvastatin within a few months prior to the detailing and sampling program. These mandatory substitution policies are very effective and do not seem to have any obvious harms [35]. Second, despite one of the program’s stated aims being to encourage switching from branded rosuvastatin to generic atorvastatin, there is very little evidence to suggest that this was an achievable goal. There have been many studies indicating physician’s reluctance to change an effective treatment [36]. Therefore, if a patient’s cholesterol was being well managed on branded rosuvastatin, a physician was unlikely to switch them to a different drug despite evidence of similar efficacy. The comparatively small impact on the choice of starting drug may have been influenced by either detailing on the part of rosuvastatin’s manufacturer or by the knowledge physicians may have had about rosuvastatin’s pending patent expiration. The data available only allowed us to evaluate the reach, fidelity, and dose of the program, but we were unable to evaluate these contextual factors that may have shaped how the intervention functioned [37].

While this study was designed to exploit the natural experiment created by the implementation of the policy, there are several limitations to our work. As our study used administrative health data, we could not examine detailed clinical data that might be obtained through medical records. Similarly, we had limited measures regarding the appropriateness of prescribing, patient satisfaction, side effects, low-density lipoprotein levels, and health outcomes for individuals who were started on one statin versus another or were switched. However, much of the available clinical evidence suggests that these drugs have similar efficacy [38]. As the generic detailing and sampling program in Manitoba was only conducted for atorvastatin, our results may not be directly applicable to other medication classes or clinical areas. However, given the widespread prescribing of these medicines, we believe our results are likely indicative of what would occur with a similar program for other popular medication classes.

## Conclusions

In a strictly regulated environment that includes generic substitution, simply releasing a low-cost generic drug will influence new starters on the drug to be prescribed the generic. Switches on the other hand tend to be more refractory, and even well-designed and evidence-based programs to accelerate these pharmaceutical policies have no room to further increase starts and do not affect switching.
